# Role of Cyclic Nucleotide Phosphodiesterases in Inner Ear and Hearing

**DOI:** 10.3389/fphys.2017.00908

**Published:** 2017-11-09

**Authors:** Rahul Mittal, Nicole Bencie, Noah Shaikh, Jeenu Mittal, Xue Zhong Liu, Adrien A. Eshraghi

**Affiliations:** Department of Otolaryngology, University of Miami Miller School of Medicine, Miami, FL, United States

**Keywords:** cyclic nucleotide phosphodiesterases, inner ear, hearing, PDE inhibitors, ototoxicity

Cyclic Nucleotide Phosphodiesterase (PDE) comprise a family of 11 enzymes that hydrolyze cyclic nucleotides. PDEs aid in the regulation of secondary messengers, cyclic adenosine monophosphate (cAMP), and cyclic guanosine monophosphate (cGMP), through the degradation of phosphodiester bonds (Huang et al., 2010; Ahmad et al., 2015; Epstein, 2017). These secondary messengers, cAMP and cGMP, are vital components of various cell signaling pathways, making their regulation by PDEs important to numerous biological processes (Weber et al., 2017; Yarwood et al., 2017). The regulation of secondary messengers by phosphodiester bond degradation occurs when PDEs hydrolyze 3′,5′ cyclic nucleotides cAMP and cGMP to their inactive nucleoside 5′-monophosphate form (Tetsi et al., 2017). While PDE functions are generally well-understood and manipulated for treatment, there exists minimal information about their role in inner ear and implications on human hearing. Though current knowledge remains limited, understanding the role of PDEs in the inner ear holds a great potential to develop novel treatment modalities for hearing loss and decrease the prevalence of deafness caused by ototoxicity.

Until recently, there was little knowledge about the expression of PDEs in the inner ear (Huang et al., 2010) and their effects on deafness. A recent study demonstrated that PDE1C, PDE4D, PDE8A, PDE9A, and PDE10A are all present in the human saccule (Degerman et al., 2017). PDE4D, PDE5, and PDE3 have all been targeted by PDE inhibitors in an attempt to treat other diseases (Turner et al., 2016; Knott et al., 2017; Maier et al., 2017; Wu et al., 2017), though these drugs are suspected to be ototoxic. PDE5 expression is observed in human saccules and it is also found in mice outer auditory sensory epithelial cells (Degerman et al., 2017). Another study demonstrated that Pde5 protein is localized in the supranuclear region below the cuticular plate in the outer hair cells (OHCs), in a dot-like pattern in the inner hair cells (IHC) synapse and in spiral ganglion neurons (SGNs) in mouse and rat cochlea (Jaumann et al., 2012). Often found in kidney cells (Ju-Rong et al., 2017), PDE4D has also been identified in human inner ear (Degerman et al., 2017). Similar to its response in the kidney, PDE4D is proposed to act as cAMP regulator responsible for controlling aqp2 translocation and vasopressin receptor type 2 responses. Known in other cell types to be induced by insulin for the facilitation of cAMP mediated triglyceride processing (Sahu et al., 2017), PDE3B is found in the apical part of the inner ear (Degerman et al., 2017).

The discovery of these various PDEs in the inner ear suggest that they play a crucial role in the auditory system (Figure 1) and can be harnessed to design novel treatment modalities. PDE4D inhibitors such as Rolipram together with brain-derived neutrophic factor (BDNF) have been shown to improve the survival rates of auditory neurons specifically SGNs in a concentration dependent manner, *in vitro* (Kranz et al., 2014). This has been attributed to the stimulation of tyrosine kinase receptor B (TrkB) that signals extracellular signal-regulated kinase (ERK)/mitogen-activated protein kinase (MAPK) pathways leading to the phosphorylation and thereby activation of cAMP-response element-binding protein (CREB; Gudasheva et al., 2013). CREB activates survival promoting genes within SGNs. In contrast, PDE5 inhibitors (Figure 1) have been associated with sudden sensorineural hearing loss in humans although larger sample size and control groups are required to confirm this association (Barreto and Bahmad, 2013). However, PDE5 inhibitor (vardenafil) has also been demonstrated to protect against noise induced hearing loss (NIHL) through activation of protective cGMP-dependent protein kinase type I (Prkg1) signaling in mouse and rat models (Jaumann et al., 2012). Thus, though it is known that PDE inhibitors have an effect on the auditory system, their exact mechanisms are much more complex than originally believed.

**Figure 1:**
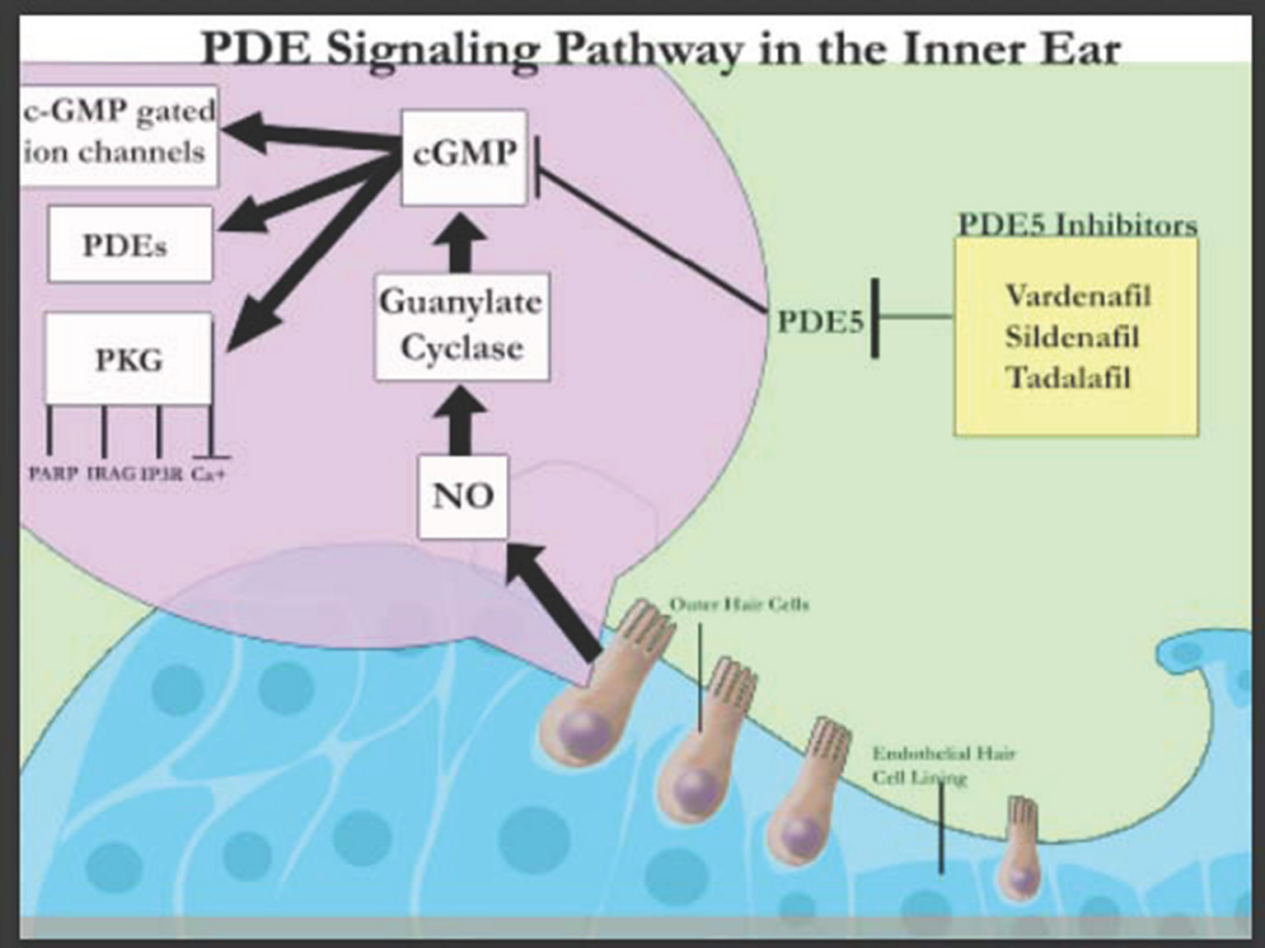
Schematic representation of PDE signaling in the inner ear (adapted from Layman and Zuo, 2012).

The future of PDE research could thus have vast implications in the field of hearing loss. Future studies should focus on deciphering the molecular mechanisms of the PDE pathways in the cochlea as well as how increasing and decreasing cAMP/cGMP levels influence inner ear homeostasis. In particular, further studying PDE5, PDE4D, and PDE3B inhibitors and their exact ototoxic properties at varying concentrations could provide insight on whether these drugs have beneficial or harmful effects on the auditory system and hearing. Once these ototoxic properties are thoroughly understood, medicinal doses can be administered or reduced accordingly. If reduction is not an option, the consequent step would involve changing the biochemical properties and structures of these PDE inhibitors to maintain their therapeutic purposes while reducing their ototoxicity.

## Author contributions

All authors listed, have made substantial, direct and intellectual contribution to the work, and approved it for publication.

### Conflict of interest statement

The authors declare that the research was conducted in the absence of any commercial or financial relationships that could be construed as a potential conflict of interest.
